# Pathogenesis of Endometriosis and Uterine Fibroids

**DOI:** 10.1155/2013/656571

**Published:** 2013-11-27

**Authors:** Pasquapina Ciarmela, Hilary Critchley, Gregory M. Christman, Fernando M. Reis

**Affiliations:** ^1^Department of Experimental and Clinical Medicine, Polytechnic University of Marche, Via Tronto 10 A, 60020 Ancona, Italy; ^2^MRC Centre for Reproductive Health, University of Edinburgh, The Queen's Medical Research Institute, 47 Little France Crescent, Edinburgh EH16 4TJ, UK; ^3^Division of Reproductive Endocrinology and Infertility, University of Florida College of Medicine, Room M302L, 1600 SW Archer Road, Gainesville, FL 32611-0294, USA; ^4^Department of Obstetrics and Gynecology, University of Minas Gerais, 30130-100 Belo Horizonte, MG, Brazil

Endometriosis and uterine fibroids are important, common pathological conditions that impose a major healthcare burden.

Endometriosis is defined as the presence of endometrial tissue outside the uterine cavity and represents one of the most frequent gynaecological disorders, affecting 10–15% of all women of reproductive age and >30% of the infertile women. 

Uterine fibroids (leiomyomas or myomas) are benign tumors of the myometrium. Uterine leiomyomas affect as many as 77% of women in reproductive age, of whom 20–50% are symptomatic.

Although they are nonneoplastic conditions, they heavily impact women's health and fertility and are a common indication for surgery, and the socioeconomic cost is huge. The mechanisms of formation remain unclear.

The knowledge and the understanding of the pathogenesis of these conditions are essential to develop successful medical therapies and to understand the mechanisms of action of the currently available therapies and are of interest to clinicians and basic and clinical researchers. 

The focus of this special issue is to highlight novel aspects pertaining to endometriosis and uterine fibroids focusing on the pathogenetic mechanisms and new avenues of enquiry (novel hypotheses).

The special issue presents reviews, research articles, and a clinical study and is the effort of a truly international group of researchers giving varied experiences from across the world. 

The paper entitled “*The natural history of uterine leiomyomas: light and electron microscopic studies of fibroid phases, interstitial ischemia, inanosis, and reclamation*” and the companion paper entitled “*The natural history of uterine leiomyomas: morphometric concordance with concepts of interstitial ischemia, inanosis*” by G. Flake et al. describe a fascinating natural history of uterine leiomyoma hypothesizing progressive developmental changes occurring in many uterine fibroids. 

The paper entitled “*Uterine fibroids: pathogenesis and interactions with endometrium and endomyometrial junction*” by A. Ciavattini et al., summarizes the available literature concerning current knowledge on pathogenesis of uterine fibroids considering risk factors, genetic, epigenetic, hormonal, and growth and differentiation contributors. This review also describes how endomyometrial junction disruption may play a crucial role in fibroid-related infertility, uterine bleeding, and growth of submucosal and intramural myomas. 

In the paper entitled “*Angiogenesis and endometriosis*” A. L. L. Rocha et al. review the evidence for the important role of angiogenesis in the pathogenesis of endometriosis and discuss the rationale for the search of antiangiogenic agents as a new therapeutic option in the treatment of endometriosis. 

In the paper entitled “*Interplay between misplaced Müllerian-derived stem cells and peritoneal immune dysregulation in the pathogenesis of endometriosis*” A. Simone Laganà et al. hypothesize that during postpubertal age, under the influence of different stimuli, misplaced and quiescent endometriotic cells derived from Müllerian structures of the embryonic female reproductive tract may acquire new phenotype, biological functions, and immunogenicity. These cells may differentiate, specializing in epithelium, glands, and stroma to form a functional ectopic endometrial tissue. 

The paper entitled “*Gene expression of leptin and long leptin receptor isoform in endometriosis: a case-control study*” is an original clinical study suggesting a putative role of leptin in the development of endometrial implants. In this study, A. P. Nácul et al. report a significantly higher serum leptin/BMI ratio in women with endometriosis as well as a significantly higher expression of leptin and long form leptin receptor transcripts in the ectopic endometrium compared to the eutopic endometrium of patients with endometriosis and those with normal pelvis (controls). 

Overall, this special issue is an excellent resource for researchers and physicians and provides “state of the art” information on these very common benign uterine/pelvic conditions, which have a major impact on women's quality of life. Although this special issue does not focus directly on therapies, this should be a valuable compendium for researchers, students, and physicians to stimulate continuing efforts to further the understanding of the pathogenesis of endometriosis and uterine fibroids and to help to develop new therapies. 

*Pasquapina Ciarmela* *Pasquapina Ciarmela* *Hilary Critchley* *Hilary Critchley* *Gregory M. Christman* *Gregory M. Christman* *Fernando M. Reis* *Fernando M. Reis*

